# THE EFFECTS OF REHABILITATION POTENTIAL ON ACTIVITIES OF DAILY LIVING IN PATIENTS WITH STROKE IN TAIWAN: A PROSPECTIVE LONGITUDINAL STUDY

**DOI:** 10.2340/jrm.v56.27028

**Published:** 2024-10-22

**Authors:** Ying-Tzu TSENG, Der-Sheng HAN, Jerry Cheng-Yen LAI, Chien-Hui WANG, Tyng-Guey WANG, Hung-Hui CHEN

**Affiliations:** 1 School of Nursing, College of Medicine, National Taiwan University, Taipei City; 2 National Taipei University of Nursing and Health Sciences, Taipei City; 3 Department of Physical Medicine and Rehabilitation, National Taiwan University Hospital, BeiHu Branch, Taipei City; 4 Department of Physical Medicine and Rehabilitation, College of Medicine, National Taiwan University, Taipei City; 5 Master Program in Biomedicine, College of Science and Engineering, National Taitung University, Taitung City; 6 Department of Nursing, National Taiwan University Hospital, Taipei City; 7 Department of Physical Medicine and Rehabilitation, National Taiwan University Hospital, Taipei City; 8 Second Degree Bachelor of Science in Nursing, College of Medicine, National Taiwan University, Taipei City; 9 Department of Nursing, National Taiwan University Hospital Hsin-Chu Branch, Hsinchu, Taiwan

**Keywords:** activities of daily living, rehabilitation potential, stroke

## Abstract

**Objective:**

This study aimed to explore the effect of three-dimensional rehabilitation potential on the activity of daily living (ADL) among patients with stroke in rehabilitation wards.

**Design:**

Prospective longitudinal study.

**Setting:**

Two rehabilitation wards situated within a nationally recognized referral centre in Northern Taiwan, followed by subsequent discharge.

**Participants:**

A total of 101 participants were admitted due to either a primary or recurring incident of infarction or haemorrhagic stroke, subsequently being transferred to the rehabilitation ward of a medical centre.

**Interventions:**

Not applicable.

**Main outcome measures:**

Rehabilitation potential included biological (swallowing ability, muscle power, and urinary incontinence), psychological (rehabilitation motivation and cognitive function), and social (social support) dimensions. The rehabilitation treatment outcome was activities of daily living measured using the Barthel Index. Time-variant variables, including swallowing ability, rehabilitation motivation, social support, and ADL, were collected at the time of transfer to the rehabilitation ward, 1–3 days before discharge, and 1 month after discharge.

**Results:**

The results of the generalized estimating equations model revealed that poor swallowing ability, lower muscle power, and urinary incontinence in the biological dimension, along with lower rehabilitation motivation and moderate cognitive impairment in the psychological dimension, are significant indicators of rehabilitation potential among stroke patients. When the different dimensional rehabilitation potential was considered overall, both biological and psychological indicators can still predict ADL outcomes during and after inpatient rehabilitation therapy. Of these indicators, swallowing ability and rehabilitation motivation were positively correlated with ADL over time. Further, increased rehabilitation motivation enhanced the protective effect of swallowing ability on ADL.

**Conclusion:**

Important indicators of rehabilitation potential, which can predict ADL outcomes, were identified for stroke patients in the rehabilitation ward. Policymakers can design appropriate intervention plans to enhance the rehabilitation potential and improve the effectiveness of inpatient rehabilitation treatment for stroke patients.

## LAY ABSTRACT

Stroke is one of the most common disorders of the central nervous system, frequently leading to functional impairments that limit patients' ability to perform activities of daily living. Rehabilitation plays a vital role in improving outcomes, particularly activities of daily living. Rehabilitation potential is a crucial predictor of therapeutic effectiveness and overall prognosis. However, despite its importance, clear measures of rehabilitation potential and its impact on outcomes such as activities of daily living remain limited. This study explores how different dimensions of rehabilitation potential, based on Mosqueda’s biopsychosocial model (biological, psychological, and socioeconomic dimensions), influence stroke patients’ activities of daily living performance during and after rehabilitation. Key findings indicate that both biological and psychological indicators predict activities of daily living outcomes, with swallowing ability and rehabilitation motivation as significant predictors. Moreover, higher rehabilitation motivation strengthens the positive effect of swallowing ability on activities of daily living. Tailored interventions could be designed to enhance rehabilitation potential and improve functional outcomes for stroke patients undergoing rehabilitation therapy.

Globally, stroke or cerebrovascular accident is the most common central nervous system disease and remains the second leading cause of death (1). The World Health Organization (WHO) reported that 15 million strokes occur annually. Of these, 5 million individuals die and another 5 million suffer from long-term disability (2). Patients with stroke usually face numerous sequelae related to functional impairments, such as limb hemiplegia, aphasia, hemianopia, visuospatial neglect, and cognitive dysfunction (2), leading to difficulties in performing activities of daily living (ADL). Motor recovery mostly occurs within 90 days after a stroke (3). Previous evidence consistently indicates that rehabilitation following a stroke should be performed as early as possible (4). Therefore, inpatient rehabilitation is crucial for improving ADL performance and reducing the length of stay (5). ADL function is the most common indicator reflecting the effectiveness of recovery in patients with stroke, during and after undergoing rehabilitation therapy (6, 7).

Rehabilitation potential anticipates the effectiveness of rehabilitation therapy, enabling patients with stroke to receive personalized therapy and aid to successfully complete their rehabilitation regimen, thereby enhancing prognostic outcomes (8). The concept of rehabilitation potential has been elucidated and explored in various studies (9–11). Mosqueda, in 1993, emphasized that rehabilitation potential should be utilized to assess a patient’s capacity to attain subsequent rehabilitation outcomes and improve their overall quality of life (9). The author further proposed a comprehensive biopsychosocial model of rehabilitation potential, encompassing aspects of the biological (cardiovascular and neuromuscular systems), psychological (rehabilitation motivation, depressive symptoms, and cognitive function), and socioeconomic dimensions (social support and insurance) (9). Moreover, it has been proposed that application of rehabilitation potential by healthcare professionals should involve consideration of time and functional recovery trajectories (10).

In recent decades, the recognition and utilization of rehabilitation potential have gradually gained prominence in both clinical practice and research (12, 13). Common criteria and principles employed to identify rehabilitation potential among patients with stroke include evaluating the level of deficits, ability to learn and participate in a rehabilitation programme, rehabilitation motivation, and psychosocial support (14, 15). However, these criteria and principles frequently lack corresponding indicators and objective metrics for evaluation (16). Moreover, the indicators chosen to predict the effectiveness of rehabilitation vary among studies when applied to patients with stroke (17–19).

Several stroke-related longitudinal studies have reported the effect of potential factors, including biological, psychological, and social factors, on prognostic outcomes, such as ADL, during and after receiving rehabilitation therapy from the inpatient to the post-discharge period (17–20). However, psychological and social factors were generally overlooked in these previous studies. Even though Mosqueda’s biopsychosocial model of rehabilitation potential has been utilized for the last 3 decades, to the best of our knowledge no quantitative studies have been conducted to explore the 3 dimensions of rehabilitation potential while concurrently examining the effects of proposed time-variant and time-invariant indicators of rehabilitation potential on ADL trajectory from the inpatient to the post-discharge period among patients with stroke. Furthermore, the interaction between the 3 dimensions of rehabilitation potential on prognostic outcomes has been less studied (21). Therefore, the goals of this study were to develop indicators of three-dimensional rehabilitation potential corresponding to Mosqueda’s biopsychosocial model (Fig. 1) and examine the relationship between rehabilitation potential and ADL trajectory from the time of transfer to the rehabilitation ward to 1 month after discharge among patients with stroke receiving inpatient rehabilitation therapy.

**Fig. 1:**
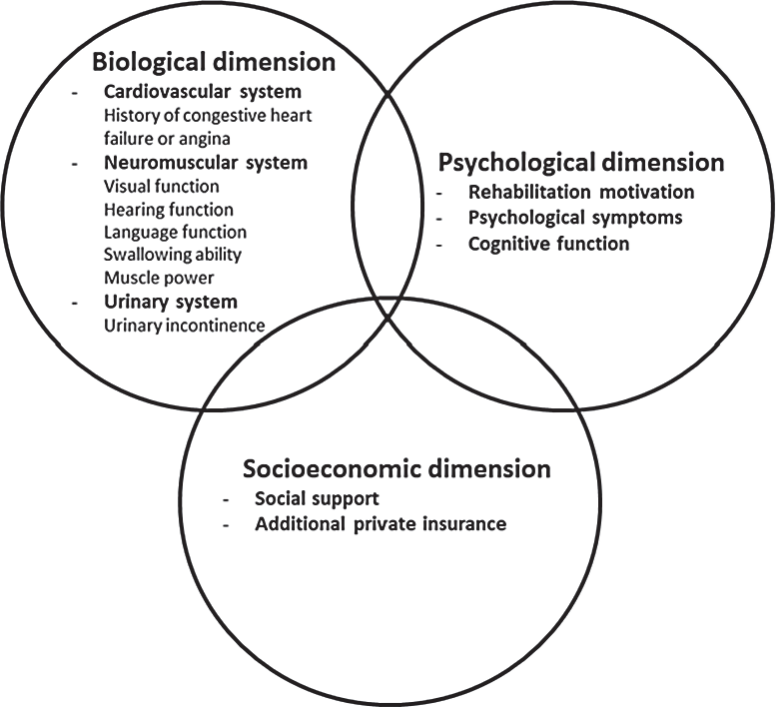
Rehabilitation potential model for the stroke population. Modified after Mosqueda9. The cardiovascular system includes a medical history of heart disorder. The neuromuscular system includes visual function, hearing function, language function, swallowing ability, and muscle power. The urinary system includes urinary incontinence.

## METHODS

This prospective longitudinal study employed a structured questionnaire completed by the participants at the time of transfer to the rehabilitation ward (the first wave), 1–3 days before discharge (the second wave), and 1 month after discharge (the third wave). The data-collection period was from January 2020 to March 2021 through face-to-face interviews in the first and second waves and subsequently through face-to-face or tele-phone interviews in the third wave at the participant’s convenience. The study protocol was approved by the Institutional Review Board of National Taiwan University Hospital, Taipei City, Taiwan (No. 201908071RINA). The study was conducted in accordance with the principles of the Declaration of Helsinki. A trained research assistant briefed the participants on the study regarding its design, purpose, and the participants’ rights. Written informed consent was obtained from all participants.

### Participants

The study population included patients with stroke who were transferred to a rehabilitation ward of a medical centre in Taipei City, Taiwan. The recruited patients with stroke were at least 20 years old and exhibited modified Rankin Scale scores of 3–5. A research assistant contacted patients with stroke in the medical centre’s rehabilitation ward and provided a brief de-scription of the purpose of the study and the participant’s rights. A signed consent form and contact information were provided by the stroke patients who agreed to participate in the study. The power and sample size for a repeated measures ANOVA F-test were calculated using G*Power software (https://www.psychologie.hhu.de/arbeitsgruppen/allgemeine-psychologie-und-arbeitspsychologie/gpower), with the following parameters: effect size f = 0.15, α = 0.05, number of groups = 1, number of measurements = 3, and a correlation among repeated measures of 0.5. The analysis determined that a minimum sample size of 73 participants was required to achieve a power of 0.8. With an actual sample size of 101, our study is likely adequately powered to detect a statistically significant difference. Of the 101 participants recruited, 98 and 93 completed the second and third interviews, respectively.

### Measurements

The study variables included background characteristics (age, sex, marital status, educational level, family income, stroke type, stroke history, stroke severity, admission days, and hospital admission rehabilitation ward days), rehabilitation potential, and ADLs. The three-dimensional rehabilitation potential comprised the following: (*i*) the biological dimension, which included the cardiovascular system (history of congestive heart failure or angina), neuromuscular system (visual neglect, hearing and language function, muscle power, and swallowing ability), and urinary system (urinary incontinence); (*ii*) the psychological dimension, including cognitive function, rehabilitation motivation, and psychological symptoms; and (*iii*) the socioeconomic dimension, including additional private insurance and social support. Among the study variables, swallowing ability, rehabilitation motivation, psychological symptoms, social support, and ADL were time-variant variables measured at the time of transfer to the rehabilitation ward, 1–3 days before discharge, and 1 month after discharge.

Language function was classified into 4 categories: (*i*) aphasia and dysarthria, (*ii*) aphasia only, (*iii*) dysarthria only, and (*iv*) none. These categories were further grouped into those with language function impairment (“aphasia and dysarthria”, “aphasia only”, and “dysarthria only”) and those with no impairment.

The swallowing ability of patients with stroke was evaluated using the Functional Oral Intake Scale, a tool that assesses the patient’s ability to swallow based on food texture and methods of food intake (22). The functional and assigned values ranged from 1 (representing non-oral intake with the use of a feeding tube) to 7 (total oral intake with no restrictions). This scale can yield continuous variables, with higher scores reflecting greater swallowing ability (23, 24).

Cognitive function was assessed using the Short Portable Mental Status Questionnaire (SPMSQ) (25). The questionnaire is composed of 10 items, including memory, orientation, attention, and calculation. An error count of 3–4 items, 5–7 items, and 8–10 items on the SPMSQ indicates the presence of mild, moderate, and severe cognitive impairment, respectively.

Rehabilitation motivation was measured using an 8-item, 4-point Likert rehabilitation motivation scale (26–28). The scale can be scored from 8 to 32 to measure the patient’s motivation for rehabilitation therapy. Higher scores indicate increased rehabilitation motivation.

Psychological symptoms were measured using the Brief Symptom Rating Scale (BSRS-5), which has been validated and used extensively in the Taiwanese population (29). BSRS-5 is composed of 5 items: sleep problems, anxiety, distress, depression, and feelings of inferiority. The BSRS-5 scale has a score range of 0–20, with higher scores indicating more severe psychological symptoms.

Social support was measured using a 9-item functional social support scale with 3 dimensions: (*i*) emotional support, which is the perceived caring, love, and empathy from others; (*ii*) instrumental support, which is the perceived support for household chores from others; and (*iii*) informational support, which is the received information on home or long-term care and assistive devices from others (30). The scale has possible scores ranging from 0 to 36. Higher scores indicate higher perceived social support.

ADL was assessed using the 10-item Barthel Index. The Barthel Index encompasses dimensions related to self-care independence (such as feeding, bathing, grooming, dressing, bowel and bladder control, and toilet use) and ADL (such as transfers from bed to chair and back, mobility on level surfaces, and stairs). The Barthel Index score ranges from 0 to 100, where a score of 100 denotes complete self-sufficiency in daily living and a score of 0 indicates total dependence on others for ADL tasks. This scale has been previously used and validated in patients with stroke (31, 32).

### Data analysis

Descriptive statistics are presented as mean, standard deviation (SD), median, range, frequency, and percentage. A generalized estimating equation (GEE) model was used to account for repeated measures. The null GEE model, which included only linear and/or quadratic terms for the time variable, was used to report the trajectories of swallowing ability, rehabilitation motivation, psychological symptoms, social support, and ADL. The GEE was also used to examine the relationship between rehabilitation potential and ADL from the time of transfer to the rehabilitation ward until 1 month after discharge. As for the significant indicators of rehabilitation potential, the effect of interactive terms of “biological and psychological/socioeconomic indicators” on ADL was examined separately due to the small cell size. Data analysis was performed using IBM SPSS Statistics (version 22.0; IBM Corp, Armonk, NY, USA).

## RESULTS

The participants’ background characteristics are summarized in Table I. Most participants were admitted on the day of stroke onset. The median number of days from hospital admission to the rehabilitation ward was 10 (range, 0–40).

**Table I:** Background variables of the study participants (*n* = 101)

Characteristic	*n*	%
Age		
< 50 years	16	15.8
≥ 50 years	85	84.2
Sex		
Female	44	43.6
Male	57	56.4
Marital status		
Single/divorced/widowed	33	32.7
Married	68	67.3
Educational level		
Junior high school or lower	36	35.6
High school or above	65	64.4
Family income^a^		
Insufficient	15	15.3
Just making a living	40	40.8
Sufficient	43	43.9
Stroke type		
Infarction	75	74.3
Haemorrhage	26	25.7
Stroke history		
No	75	74.3
Yes	26	25.7
Onset stroke severity (NIHSS)^b^		
0–6	30	38.5
7–15	28	35.9
> 15 (worse)	20	25.6

a *n* = 98;

b *n* = 78.

**Fig. 2:**
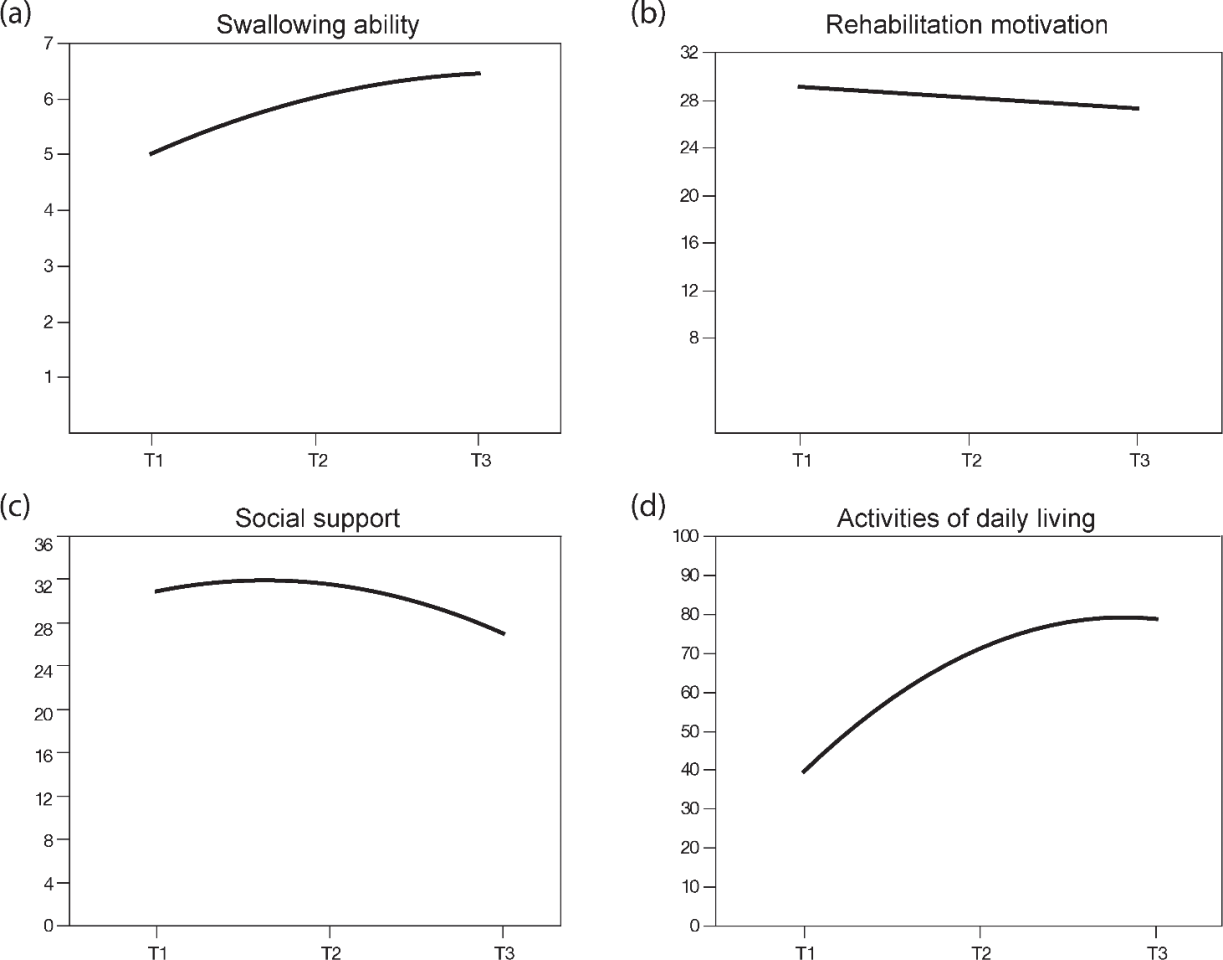
Time trends of swallowing ability, rehabilitation motivation, social support, and activities of daily living from admission to the rehabilitation ward to 1 month after discharge. T1: Admission to the rehabilitation ward; T2: 1–3 days before discharge; T3: 1 month after discharge.

The rehabilitation potential and ADL of the study participants are summarized in Table II. The mean muscle power in the lower limbs of the affected side in patients with stroke was 3.53 ± 1.07. Over 40% of the participants had urinary incontinence and approximately 15% had moderate cognitive impairment, with no cases of severe cognitive impairment observed. The mean scores for swallowing ability and ADL increased, whereas the mean score for rehabilitation motivation decreased from the time of transfer to the rehabilitation ward until 1 month after discharge. A null GEE model was used to examine the trajectories of the aforementioned time-varying variables. In the models for rehabilitation motivation, only the linear terms were significant. Rehabilitation motivation followed a downward linear trajectory from the time of transfer to the rehabilitation ward to 1 month after discharge (Fig. 2). Both the linear and quadratic terms were significant in the swallowing ability, social support, and ADL models. The scores for swallowing ability and ADL were lowest at the time of transfer to the rehabilitation ward, increased sharply until 1–3 days before discharge, and then gradually flattened between 1–3 days before discharge and 1 month after discharge; the score for social support slightly increased from the time of transfer to the rehabilitation ward to 1–3 days before discharge, and then decreased sharply until 1 month after discharge (Fig. 2).

**Table II:** Rehabilitation potential and activities of daily living of the study participants (*n* = 101)

Variables	Rehabilitation potential†							
	Biological dimension						Psychological dimension	Socio-economic dimension
	Cardiovascular system	Neuromuscular system				Urinary system		
	History of congestive heart failure or angina *n* (%)	Visual neglect *n* (%)	Hearing impairment *n* (%)	Language function impairment^f^ *n* (%)	Muscle power in the lower limb of the affected side Mean (SD)	Urinary incontinence *n* (%)	Moderate cognitive impairment *n* (%)	Additional private insurance *n* (%)
T1: Admission to the rehabilitation ward	19 (18.8%)	11 (14.1%)^a^	8 (7.9%)	46 (45.5%)	3.53 (1.07)^b^	44 (43.6%)	17 (16.8%)	31 (31.6%)^c^

**Table:** 

Variables	Rehabilitation potential††				Activities of daily living^††^ Mean (SD)
	Biological dimension	Psychological dimension		Socioeconomic dimension	
	Neuromuscular system				
	Swallowing ability Mean (SD)	Rehabilitation motivation Mean (SD)	Psychological symptoms Mean (SD)	Social support Mean (SD)	
T1: Admission to the rehabilitation ward	5.02 (2.39)	28.91 (2.07)	3.78 (4.24)	30.92 (3.56)	39.65 (23.67)
T2: 1–3 days before discharge^c^	6.05 (1.59)	28.77 (2.86)	3.10 (3.70)	31.54 (4.40)	71.33 (23.92)
T3: 1 month after discharge^b^	6.53 (1.02)^d^	27.07 (3.78)^e^	3.16 (3.81)	26.99 (10.63)	80.05 (26.37)
GEE null model	β (SE)	β (SE)	β (SE)	β (SE)	β (SE)
Linear model					
Intercept	5.07 (0.23)***	29.12 (0.19)***	3.71 (0.41)***	31.70 (0.41)***	41.18 (2.31)***
Linear	0.73 (0.11)***	–0.91 (0.20)***	–0.31 (0.25)	–1.92 (0.59)**	19.87 (1.13)***
Quadratic model					
Intercept	5.02 (1.30)***			30.92 (0.35)***	39.65 (2.34)***
Linear	1.30 (0.27)***			3.23 (0.96)***	43.57 (2.89)***
Quadratic	–0.29 (0.10)**			–2.59 (0.62)***	–12.00 (1.25)***

a *n* = 78,

b *n* = 93,

c *n* = 98,

d *n* = 92,

e *n* = 87;

f language function impairment includes aphasia or difficulty in articulation.

† Baseline characteristics of the rehabilitation potential at admission (T1).

†† Rehabilitation potential and activities of daily living of the study participants and their null model of trajectories from admission to the rehabilitation ward (T1) to 1 month after discharge (T3). Working correlation matrix: AR(1).

SD: standard deviation.

** *p* < 0.01;

*** *p* < 0.001.

The effect of rehabilitation potential indicators on ADL from the time of transfer to the rehabilitation ward to 1 month after discharge are presented in Table III. In Models 1–4, both linear and quadratic terms were significant, indicating that ADL followed an upward curvilinear trajectory. The 3 dimensions of rehabilitation potential were considered separately in Models 1–3, and the significant indicators of the three-dimensional rehabilitation potential in these 3 models were further analysed in Model 4. However, no indicators of the socioeconomic dimension were significantly associated with ADL in the socioeconomic dimension models (Model 3). When the significant biological and psychological indicators of rehabilitation potential were considered concurrently (Model 4), swallowing ability (β = 3.19, standard error [SE] = 0.72), muscle power in the lower limb of the affected side (β = 7.34, SE = 1.16), and urinary incontinence (β = –15.49, SE = 2.56) in the biological dimension, rehabilitation motivation (β = 1.30, SE=0.29), and moderate cognitive impairment (β = –8.59, SE = 2.65) were significantly associated with ADL. Patients with high swallowing ability, muscle power, and rehabilitation motivation had higher ADL scores. In contrast, patients with stroke who had urinary incontinence and moderate cognitive impairment experienced lower levels of ADL. Patients with stroke aged 50 years had lower ADL scores.

**Table III:** GEE Model for ADL trajectory from admission to the rehabilitation ward to 1 month after discharge

Variable	Unadjusted model		Adjusted model							
			Model 1		Model 2		Model 3		Model 4	
			Model of biological dimension		Model of psychological dimension		Model of socioeconomic dimension		Model of 3 dimensions	
	β (SE)	95% CI	β (SE)	95% CI	β (SE)	95% CI	β (SE)	95% CI	β (SE)	95% CI
Trend over time										
Linear (time)			40.81 (3.38)***	[34.20, 47.43]	42.62 (2.75)***	[36.23, 47.02]	42.91 (2.92)***	[37.19, 48.64]	38.62 (2.94)***	[32.85, 43.39]
Quadratic			–11.28 (1.49)***	[–14.19, –8.37]	–10.26 (1.23)***	[–12.67, –7.85]	–11.60 (1.27)***	[–14.08, –9.11]	–9.90 (1.29)***	[–12.43, –7.37]
Background variable										
Age: ≥ 50 years old			–4.39 (2.58)	[–9.44, 0.66]	–14.20 (3.79)***	[–21.64, –6.77]	–15.14 (4.57)**	[–24.10, –6.18]	–9.37 (2.85)**	[–14.95, –3.78]
Sex: male			4.85 (2.63)	[–0.31, 10.01]	5.41 (3.63)	[–1.71, 12.53]	6.01 (4.30)	[–2.43, 14.44]	2.48 (2.29)	[–2.01, 6.96]
Biological dimension										
History of congestive heart failure or angina	–8.58 (6.42)	[–21.16, 4.00]	–0.13 (2.57)	[–5.17, 4.91]						
Visual neglect	–20.67 (8.62)*	[–37.57, –3.76]	–0.22 (4.59)	[–9.21, 8.77]						
Hearing impairment	–9.67 (4.68)*	[–18.85, –0.50]	0.37 (1.49)	[–2.56, 3.30]						
Language function impairment	–10.05 (4.55)*	[–18.97, –1.12]	–2.61 (2.54)	[–7.59, 2.37]						
Swallowing ability	9.31 (0.74)***	[7.86, 10.76]	3.13 (1.02)**	[1.13, 5.14]					3.19 (0.72)***	[1.79, 4.60]
Muscle power in the lower limb of the affected side	12.84 (1.74)***	[9.44, 16.25]	8.51 (1.83)***	[4.94, 12.09]					7.34 (1.16)***	[5.06, 9.61]
Urinary incontinence	–28.76 (3.82)***	[–36.26, –21.27]	–17.29 (3.60)***	[–24.34, –10.24]					–15.49 (2.56)***	[–20.51, –10.48]
Psychological dimension										
Rehabilitation motivation	0.96 (0.42)*	[0.13, 1.80]			1.40 (0.31)***	[0.78, 2.01]			1.30 (0.29)***	[0.72, 1.84]
Psychological symptoms	–1.06 (0.40)**	[–1.84, –0.27]			–0.23 (0.31)	[–0.84, 0.37]				
Moderate cognitive impairment	–21.35 (5.25)***	[–31.64, –11.05]			–11.97 (3.60)**	[–18.97, –4.97]			–8.59 (2.65)**	[–13.79, –3.39]
Social dimension										
Social support	1.13 (0.17)***	[0.79, 1.47]					0.08 (0.13)	[–0.17, 0.33]		
0Additional private insurance	–0.22 (5.00)	[–10.02, 9.59]					–1.11 (4.81)	[–10.53, 8.31]		

Working correlation matrix: AR (1). GEE: generalized estimating equation; SE: standard error; CI: confidence interval.

* *p* < 0.05;

** *p* < 0.01;

*** *p* < 0.001.

The GEE reported that the interaction between swallowing ability (biological dimension) and rehabilitation motivation (psychological dimension) on ADL was significant (Table IV). Swallowing ability and motivation for rehabilitation were dichotomized as categorical variables. Compared with stroke patients who had low levels of swallowing ability and rehabilitation motivation, those who had low levels of swallowing ability and high levels of rehabilitation motivation (β = 18.38, SE = 4.06, *p* < 0.001), high levels of swallowing ability and low levels of rehabilitation motivation (β = 28.65, SE = 4.54, *p* < 0.001), and high levels of both swallowing ability and rehabilitation motivation (β = 30.10, SE = 4.16, *p* < 0.001), had higher levels of ADL.

**Table IV:** Generalized estimating equation models of the effect of the interaction between biological (swallowing ability) and psychological (rehabilitation motivation) indicators of rehabilitation potential on activities of daily living

Model	Activities of daily living		
	β (SE)	95% CI	*p*-value
Swallowing ability^a^ and rehabilitation motivation^b^			
Low level of swallowing ability, low level of rehabilitation motivation	1		
Low level of swallowing ability, high level of rehabilitation motivation	18.38 (4.06)	[10.41, 26.35]	< 0.001***
High level of swallowing ability, low level of rehabilitation motivation	28.65 (4.54)	[19.76, 37.54]	< 0.001***
High level of swallowing ability, high level of rehabilitation motivation	30.10 (4.16)	[21.95, 38.25]	< 0.001***

Working correlation matrix: AR (1).

The adjusted model included time (before vs after receiving hospital rehabilitation), age (< 50 years old vs ≥ 50 years old), and sex (female vs male).

a Cut-off point of swallowing ability: 3/4.

b Cut-off point of rehabilitation motivation: 28/29 [median].

SE: standard error; CI: confidence interval.

*** *p* < 0.001.

## DISCUSSION

According to the study’s findings, ADL during and after inpatient rehabilitation therapy followed an upward curvilinear trajectory. The key indicators of rehabilitation potential for ADL outcomes among stroke patients were primarily from the biological dimension (e.g., swallowing ability) and the psychological dimension (e.g., rehabilitation motivation), with no significant indicators from the socioeconomic dimension. Of the significant indicators of rehabilitation potential, both swallowing ability and rehabilitation motivation changed over time. Moreover, the interaction between swallowing ability and rehabilitation motivation was significant, indicating that the effects on ADL during and after inpatient rehabilitation therapy varied among those who had different levels of swallowing ability with different levels of rehabilitation motivation.

ADL scores among patients with stroke were the lowest before undergoing inpatient rehabilitation therapy in the rehabilitation ward, increased sharply until 1–3 days before discharge, and then gradually increased slightly between 1–3 days before discharge and 1 month after discharge. This upward curvilinear trajectory was concurrent with previous stroke studies showing that the ADL trajectory drastically increased initially and then increased to a lesser extent thereafter (33, 34). Previous evidence has shown that the 90-day period following stroke is the main period for functional recovery (3). Moreover, inpatient rehabilitation performed in the early post-stroke period can significantly promote functional recovery. These reasons could explain why ADL initially increased sharply in patients with stroke.

Rehabilitation potential may change over time (10). Of the significant time-variant indicators of rehabilitation potential regarding ADL, the swallowing ability and rehabilitation motivation changed over time in this study. Swallowing ability followed an upward curvilinear trajectory. The increased change in swallowing status during the inpatient period was greater than that in the early post-discharge period, which may be related to the effectiveness of inpatient swallowing therapy for stroke patients with dysphagia (35, 36). The study found that rehabilitation motivation slightly decreased from admission to the rehabilitation ward to 1 month after discharge. This study was conducted during the coronavirus disease 2019 (COVID-19) outbreak, when hospitals in Taiwan imposed strict visitor restrictions. At our hospital, we limited the number of visitors/caregivers to 1 per patient. During this period, patients with stroke tended to be homesick and wanted to recover quickly. Subsequently, they received assistance, encouragement, and follow-up from the inpatient rehabilitation team, which could have enhanced their motivation for rehabilitation. Taken together, patients with stroke reported increased rehabilitation motivation and perceived better social support from the healthcare team during the inpatient period compared with the post-discharge period.

Both biological and psychological indicators of rehabilitation potential are critical factors related to the functional effectiveness of rehabilitation. To the best of our knowledge, this is the first longitudinal study that attempted to delineate the effects of the different sub-dimensions of rehabilitation potential on ADL. When statistically significant indicators from the 3 dimensions of rehabilitation potential were considered together, these biological and psychological indicators were still significantly associated with ADL among patients with stroke in the rehabilitation ward. The findings of this investigation imply that individual biological and psychological attributes play a more prominent role in determining the efficacy of rehabilitation compared with socioeconomic indicators (social support and additional private insurance). However, this result may be influenced by the specific socioeconomic indicators selected for analysis. Notably, stroke patients in this study reported higher levels of social support, likely due to long-term care resources and universal health insurance provided by government policies, along with assistance from healthcare teams and familial networks. The relatively low variation in social support among stroke patients makes it more challenging to reflect the correlation between social support and ADL outcomes, despite previous studies indicating an association between the two (37–39). Additionally, the widespread universal health coverage in Taiwan could potentially diminish the differential advantages conferred by private insurance on the functional outcomes of stroke patients. Although our findings did not identify significant indicators of rehabilitation potential from the socioeconomic dimension, the possibility of alternative indicators cannot be ruled out. Given the differences in sociocultural environments, future research could incorporate relevant socioeconomic indicators for further investigation.

Mosqueda’s rehabilitation potential model demonstrated the convergence of 3 (biological, psychological, or social) dimensions, which implied that the interaction among the 3 dimensions should be considered.^9^ Our findings on the effects of the interaction between the biological dimension and psychological dimension on ADL could be evidence to support Mosqueda’s model. This study indicated that a disparity between the connection between swallowing ability (biological dimension) and ADL differed depending on the level of rehabilitation motivation (psychological dimension). A stronger relationship between swallowing ability and ADL was observed in patients with stroke with higher rehabilitation motivation. Strategies to increase rehabilitation motivation should be developed to enhance the effect of swallowing ability on ADL in patients with stroke.

Over the past 3 decades, rehabilitation potential has garnered sustained attention; however, a unified and standardized method for its measurement has yet to be established. While previous literature has primarily focused on conceptual definitions of rehabilitation potential, only a limited number of studies have addressed operational definitions and measurement frameworks (9–11). Among these, Mosqueda’s three-dimensional biopsychosocial model emerges as a notable framework for measuring rehabilitation potential (9). In this quantitative study, corresponding measurement indicators of rehabilitation potential were developed based on Mosqueda’s model to predict the effectiveness of rehabilitation. Future research could examine the impact of these indicators on other rehabilitation outcomes, such as quality of life. Moreover, objective criteria for assessing rehabilitation potential should be established, and scales could be further developed to enhance the evaluation of rehabilitation potential. Furthermore, machine-learning techniques can be integrated into clinical practice to provide healthcare professionals with a quick and straightforward assessment of rehabilitation potential.

### Limitations

This study has some limitations. Among the patients with stroke who participated in this study, no statistically significant differences were found between those who completed the 3-wave questionnaire and those who did not. To increase response rates, we used 3 different modes (face-to-face interview, telephone interview, and email) to collect data for practical reasons with low attrition rates (2.97% in the second wave and 5.10% in the third wave). However, the mode effect cannot be ruled out in the data between the first and the third wave. All patients with stroke in this study were recruited from single medical centre. However, this medical centre is the largest national referral centre in Taiwan. Additionally, certain factors should be considered when studying the link between rehabilitation potential and outcomes. Life goals, which are the “desir-ed states that individuals seek to achieve, maintain, or avoid”, can influence rehabilitation motivation and in turn may affect the rehabilitation outcomes (40). These goals might shape why someone pursues rehabilitation and how much recovery they aim for. Therefore, future studies should take into account the reasons for pursuing rehabilitation, the desired level of recovery, and life goals when exploring the relationship between rehabilitation potential and outcomes. Lastly, because various medical systems, welfare policies, and family cultures largely vary in different countries, it is yet to be determined whether the findings can be generalized to patients with stroke with different sociocultural backgrounds, hence meriting further study.

### Conclusions

The period from receiving inpatient rehabilitation therapy to 1 month after discharge is a crucial phase for the recovery of ADL function. To improve ADL performance, appropriate strategies should be developed according to the rehabilitation potential of patients with stroke. A rehabilitation potential checklist for functional outcome could be built upon Mosqueda’s biopsychosocial model of rehabilitation potential, consisting of biological (swallowing ability, muscle power, and urinary incontinence) and psychological (rehabilitation motivation and cognitive function) indicators, which could be used to detect objective levels of rehabilitation potential early on. Additionally, swallowing ability and rehabilitation motivation are dynamic and time-variant indicators of rehabilitation potential during the inpatient and post-discharge periods. Policymakers and health practitioners should pay more attention to rehabilitation potential status over time and provide corresponding interventions to improve post-stroke ADL function.
